# Arabic validation of the incharge financial distress/financial well-being scale and the new single-item financial stress scale

**DOI:** 10.3389/fpubh.2025.1570404

**Published:** 2025-06-09

**Authors:** Christian-Joseph El Zouki, Abdallah Chahine, Rabih Hallit, Diana Malaeb, Sami El Khatib, Antonio Nehme, Sahar Obeid, Feten Fekih-Romdhane, Souheil Hallit

**Affiliations:** ^1^UFR de Médecine, Université de Picardie Jules Verne, Rue des Louvels, Amiens, France; ^2^School of Medicine and Medical Sciences, Holy Spirit University of Kaslik, Jounieh, Lebanon; ^3^Department of Infectious Disease, Bellevue Medical Center, Mansourieh, Lebanon; ^4^Department of Infectious Disease, Notre Dame des Secours, University Hospital Center, Byblos, Lebanon; ^5^College of Pharmacy, Gulf Medical University, Ajman, United Arab Emirates; ^6^Department of Biomedical Sciences, School of Arts and Sciences, Lebanese International University, Bekaa, Lebanon; ^7^Center for Applied Mathematics and Bioinformatics (CAMB), Gulf University for Science and Technology (GUST), Hawally, Kuwait; ^8^Department of Social and Education Sciences, School of Arts and Sciences, Lebanese American University, Jbeil, Lebanon; ^9^Department of Psychiatry "Ibn Omrane", The Tunisian Center of Early Intervention in Psychosis, Razi Hospital, Maouba, Tunisia; ^10^Faculty of Medicine of Tunis, Tunis El Manar University, Tunis, Tunisia; ^11^Department of Psychology, College of Humanities, Effat University, Jeddah, Saudi Arabia; ^12^Applied Science Research Center, Applied Science Private University, Amman, Jordan

**Keywords:** incharge financial distress/financial well-being scale, financial stress, Arabic, factor analysis, validation

## Abstract

**Background:**

The current study aimed to investigate the factor structure, reliability, and validity of the Arabic adaptation of the InCharge Financial Distress/Financial Well-Being Scale, and to examine a newly developed single-item measure of financial stress, the Single-Item Financial Stress scale (SIFiS), in a sample of Lebanese adults.

**Methods:**

In this cross-sectional study, 403 participants completed an Arabic-translated version of the IFDFW scale via an online survey. Confirmatory factor analysis (CFA) was used to validate the scale.

**Results:**

A one-factor structure was supported by the analysis. Internal reliability was excellent, with very high omega and alpha coefficients for the IFDFW scale (*ω* = 0.95, *α* = 0.95). A significantly lower mean IFDFW score was found in males compared to females. On the other hand, no significant differences were found between males and females on the SIFiS scores. Greater financial burden was significantly associated with higher levels of depression, anxiety, and stress.

**Conclusion:**

The findings confirm that the Arabic versions of the IFDFW scale and the SIFiS are valid and reliable. Their use is therefore recommended in various settings among Arabic-speaking adults. These simple and straightforward measurement tools may improve cross-cultural studies on financial well-being.

## Background

Financial wellness has gained increasing attention among policymakers, economists, and mental health professionals. It is commonly defined as “the perception of being able to sustain current and anticipated desired living standards and financial freedom” (1). However, financial well-being is susceptible to financial distress, which refers to the perceived financial strain and negative emotional responses to one’s financial situation, such as stress, worry about expenses, and dissatisfaction with financial stability (2). Financial well-being is shaped by the interaction of personal, contextual, and behavioral factors that influence an individual’s financial experiences. These include one’s income (3), education (4), and employment stability (5), as well as behavioral financial practices like saving, budgeting (6), and credit management (7), which are associated with satisfaction. In contrast, impulsive spending and overborrowing have been linked to increased financial stress (8, 9). Contextual elements, such as economic conditions and inflation, also affect one’s ability to manage finances competently (10). Beyond its economic implications, financial stress is closely linked to mental health, as it can worsen anxiety and depression, and is increasingly recognized as a key social determinant of health influencing both psychological and physical outcomes (11–13).

Financial well-being is commonly assessed using both objective indicators (income, savings, debt) and subjective perceptions, such as control, security, and satisfaction. Recent research (1, 14, 15) suggests that subjective measures often outperform traditional metrics as they better reflect individuals’ lived experiences. These perceptions vary widely, even among those in similar financial situations. For instance, a high-income individual with heavy expenses may feel less financially secure than someone with modest means but greater stability. This growing emphasis on subjective assessment reflects the need for tools that capture the complexity and personal nature of financial well-being across diverse contexts.

The InCharge Financial Distress/Financial Well-Being (IFDFW) Scale (16), developed by Prawitz et al. through a Delphi study and rigorous statistical testing, is a widely used tool for assessing financial well-being. It consists of eight self-report items rated on a 10-point Likert scale continuum, measuring both financial stress and satisfaction. Higher scores indicate better perceived control and well-being, while lower scores reflect financial distress and insecurity. Example items include: “How do you feel about your current financial situation?” and “How frequently are you concerned about whether you can pay for customary monthly living expenses?.” The original validation paper supported a one-factor structure, with strong item loadings (0.83–0.93) and excellent internal consistency (Cronbach’s *α* = 0.95).

To ensure its cross-cultural relevance, the IFDFW Scale has been adapted into several languages, including Malay (17), Brazilian Portuguese (18), German, Italian, Dutch, Slovenian, and Spanish (19). These adaptations consistently supported the original one-factor structure and demonstrated strong psychometric properties. Internal consistency was excellent across most versions (Cronbach’s *α* > 0.90). Fit indices such as the Comparative Fit Index (CFI) and Standardized Root Mean Square Residual (SRMR) were consistently excellent, while the Tucker-Lewis Index (TLI) ranged from acceptable to excellent. However, the Root Mean Square Error of Approximation (RMSEA) values varied: they were excellent in Slovenia, the United Kingdom, and the United States; good in Spain, but less satisfactory in Italy, Germany, and the Netherlands. Despite these variations, the scale demonstrated overall validity and reliability across diverse cultural contexts.

The IFDFW Scale has been widely applied in research across disciplines, particularly in psychology and health, to examine associations between financial well-being and outcomes like mental health (20–22) and quality of life (23). It is valued for being time-efficient, easy to administer, and adaptable to diverse populations and settings. A recent systematic review (24) highlighted the IFDFW among the top financial well-being instruments based on its robust psychometric properties. Its strong internal consistency and structural validity have been consistently supported in multiple studies (2, 17, 19). Validating the IFDFW Scale in Arabic within the Lebanese context is particularly important given the severity of financial stress in this region. Between 2012 and 2022, the proportion of individuals living below the poverty line in Lebanon rose from 12 to 44% (25). Inflation reached a historic high of 221.3% in 2023 (26), and the Lebanese pound has lost over 98% of its value since 2019 (27), making it one of the world’s weakest currencies. These conditions have intensified financial strain and its psychological consequences. Despite the scale’s frequent use in Lebanese studies (28–32), no comprehensive psychometric validation of the IFDFW has been conducted in Arabic. Addressing this research gap is essential to ensure the scale’s conceptual equivalence, cultural relevance, and measurement accuracy in Arabic-speaking populations.

In addition to validating the Arabic version of the IFDFW, the second objective of this study was to develop and validate a single-item measure of financial stress, which we named the Single-Item Financial Stress Scale (SIFiS). Single-item self-report tools offer significant practical advantages: they are brief, easy to administer and cost-effective. Such measures are particularly valuable in large-scale field research or time-constrained environments, where reducing participant burden and overall study expenses is essential. Beyond their practicality, single-item scales have shown good validity and reliability and are increasingly recognized in recent guidelines (33, 34).

Single-item tools have already been successfully used in various psychological and health domains, including job satisfaction (35), social identity (36), and financial toxicity (37). Likewise, they have been applied in financial well-being studies. A US-based study on homeowners (38) used a single question to assess financial satisfaction, and an Australian study (39) conducted during the COVID-19 pandemic followed a similar approach. An Italian investigation (40) on emerging adults employed single-item measures to assess both financial well-being and stress. For this study, we adapted item 8 from the IFDFW Scale, which measures general financial stress, as the basis for the SIFiS. This choice is supported by the Italian study (40), which demonstrated that daily objective financial stress—unlike daily objective financial well-being—significantly impacts both subjective financial well-being and subjective financial stress. This is due to negativity bias (41, 42), whereby economic losses weigh more heavily on perceptions than economic gains. Moreover, the Italian study (40) emphasized that the choice between measures of financial stress and financial satisfaction should be context-specific. Given Lebanon’s prolonged economic crisis and the mental health focus of this study, we prioritized financial stress as a more appropriate and sensitive marker. The most comparable precedent to our SIFiS is a US college study (43), where participants were asked “How stressed do you feel about your personal finances?” and responded using a 10-point scale. By validating a similar single-item measure in Arabic, we aim to fill a key research gap in the availability of brief, culturally adapted tools to assess financial stress in Arabic-speaking populations.

Therefore, the aim of this study was two-fold: (1) to translate and validate the Arabic version of the IFDFW Scale within a sample of Arabic-speaking Lebanese adults, and (2) to develop and test the psychometric properties of a single-item scale measuring financial stress (i.e., the SIFiS). We hypothesized that the Arabic version of the IFDFW scale would demonstrate: (1) strong internal consistency, (2) a well-fitting factor structure, (3) measurement invariance across sex, and (4) convergent validity, evidenced by correlations between the IFDFW score and depression, anxiety, and stress scores. We also anticipated that the SIFiS would demonstrate similar psychometric patterns, supporting its utility as a brief measure of financial stress.

## Methods

### Study design and participants

This cross-sectional study was conducted between September and October 2021 using an anonymous online questionnaire created on Google Forms and disseminated to a sample of Lebanese adults from across the country. Eligible participants were Lebanese citizens aged 18 or older who gave consent to participate. Participants were recruited via snowball sampling through online platforms and social media applications. Prior to participation, the survey’s objectives and general instructions were clearly explained.

The survey was prepared in Arabic and consisted of three sections. The first section consisted of an online consent checkpoint to ensure voluntary participation, and attention to ethical concerns, such as confidentiality and anonymity of responses. This section also provided an introduction to the study and instructions on how to complete the questionnaire. The second section collected sociodemographic information, such as sex, age and socioeconomic status (SES); the latter was assessed through the household crowding index (HCI), calculated by dividing the number of persons by that of the rooms in the house without the kitchen and bathrooms (44). In the third section, validated measures were introduced, as outlined in the following details.

### Measures

#### Incharge’s financial distress/financial well-being scale

This scale was designed to measure a person’s sense of security or distress regarding their financial situation (2). It consists of 8 items rated on a scale from 1 to 10. A higher score indicates lower perceived pressure and an improved state of personal financial wellness. Written permission for its use has been obtained from the original authors. The current Cronbach’s alpha is 0.95.

#### The single-item financial stress scale

Item 8 of the IFDFW was selected and analyzed separately as a potential standalone measure for assessing financial stress. The item is worded as follows: “How stressed do you feel about your personal finances in general?” (2). Responses are rated on a 10-point scale ranging from 1 (“Overwhelming Stress”) to 10 (“No Stress at All”).

#### Lebanese anxiety scale (LAS-10)

This 10-item instrument (45) was specifically developed to assess symptoms of anxiety within a Lebanese setting. The first seven questions are rated from 1 to 10, and the last three questions are based on the frequency of symptom manifestation, rated from 1 to 4. Higher total scores indicate higher anxiety. The current Cronbach’s alpha value is 0.89.

#### Beirut distress scale (BDS-10)

This scale was developed and validated in the Lebanese context using a 10-item questionnaire measuring psychological distress (46). The response format is a 4-point Likert scale ranging from 0 (never) to 4 (always), where higher scores reflect greater psychological distress. The current Cronbach’s alpha is 0.90.

#### Patient health questionnaire (PHQ-9)

This scale is among the most widely used and validated tools for assessing depression (47). It is a 9-item self-administered questionnaire, already validated in Lebanon (48). Each item is rated from 0 (“not at all”) to 3 (“nearly every day”). Higher scores indicate greater levels of depression. The current Cronbach’s alpha is 0.90.

### Translation procedure

Forward and backward translations were performed to achieve a valid Arabic translation of the IFDFW. First, an independent Lebanese translator translated the English version of the IFDFW into Arabic. Then, a Lebanese academic with proficiency in both languages back-translated the Arabic version into English. A bilingual expert panel—composed of the research team and two professional translators—carefully reviewed and compared the original and back-translated English versions to evaluate both conceptual and linguistic accuracy. Discrepancies were discussed and resolved by consensus to ensure equivalence. Finally, the clarity and comprehensibility of the items were assessed in a pilot study with 30 participants, which confirmed that no further modifications were necessary.

### Minimal sample size calculation

We calculated a minimum sample size of 160 participants, following the guideline of 20 participants per item in the scale (49).

### Analytic strategy

A confirmatory factor analysis (CFA) was performed using SPSS AMOS, version 28. Parameter estimation was based on the maximum likelihood method. Several fit indices were calculated: the Root Mean Square Error of Approximation (RMSEA ≤ 0.08), Standardized Root Mean Square Residual (SRMR ≤ 0.05), Tucker-Lewis Index (TLI), and Comparative Fit Index (CFI; both ≥ 0.90) (50). For convergent validity, the average extracted variance (AVE) was considered for which the threshold value is ≥ 0.50 (51). Since multivariate normality was not supported at the beginning based on Bollen-Stine bootstrap *p* = 0.002, non-parametric bootstrapping approach was conducted.

Measurement invariance across gender of the IFDFW scores was tested through multi-group CFA (52), at configural, metric and scalar invariance levels (53). Evidence of invariance was supported when ΔCFI ≤ 0.010 and ΔRMSEA ≤ 0.015 or ΔSRMR ≤ 0.010 (52, 54). Comparisons of IFDFW and SIFiS scores across sexes were tested using the Student *t*-test.

SPSS v.27 software was used to conduct the remaining analysis. Composite reliability was determined using McDonald’s *ω* and Cronbach’s *α*, with >0.70 being considered acceptable reliability (55, 56). Skewness and kurtosis values between −1 and +1 indicated that SIFis scores were normally distributed (57). Associations between IFDFW and SIFiS scores and other variables were evaluated using the Pearson correlation test. We conducted a Receiver Operating Characteristics (ROC) analysis to confirm the suitability of the SIFiS as a standalone measure, by dichotomizing the total IFDFW score, using ≤ 40 as a threshold to define high financial stress cases. Criterion validity was evaluated via the calculation of the sensitivity and specificity values.

## Results

### Participants

A total of 403 participants were enrolled in this study, with a mean age of 32.76 ± 13.24 years, and 65.5% were females. Additional demographic details are presented in Table 1.

**Table 1:** Sociodemographic characteristics of the participants (*N* = 403).

Variable	N (%)
Sex	
Males	139 (34.5%)
Females	264 (65.5%)
Marital status	
Single / divorced / widowed	266 (66.0%)
Married	137 (34.0%)
Education level	
Secondary or less	68 (16.9%)
University	335 (83.1%)
	Mean ± SD
Age (in years)	32.76 ± 13.24
Household crowding index (person/room)	1.05 ± 0.51

### Confirmatory factor analysis

**Figure 1:**
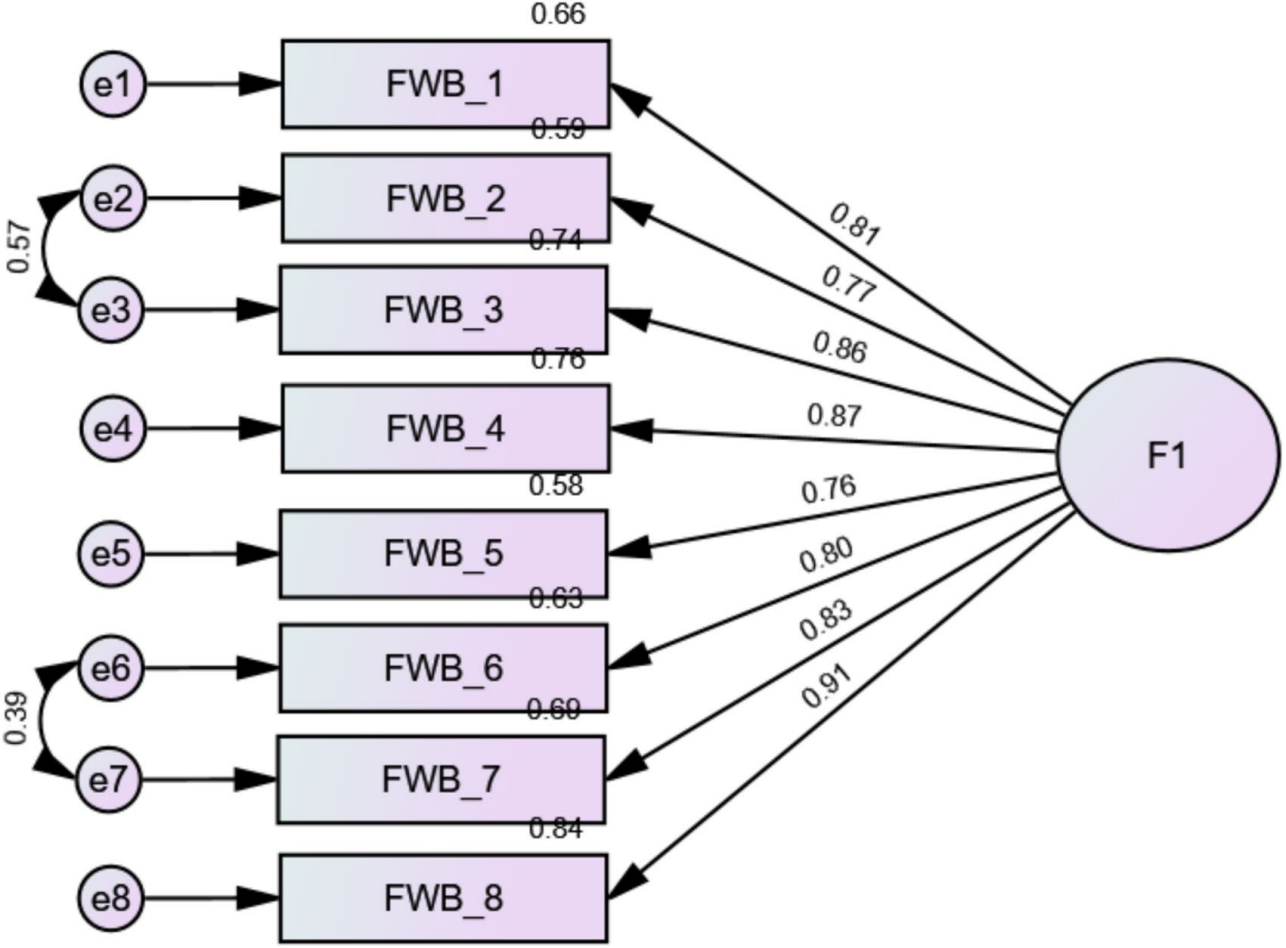
Standardized loading factors deriving from the confirmatory factor analysis of the incharge financial distress/financial well-being scale.

The fit indices were acceptable [RMSEA = 0.164 (90% CI 0.146, 0.183), SRMR = 0.039, CFI = 0.926, TLI = 0.897] but improved after adding correlations between items 2–3 and 6–7 due to high modification indices [RMSEA = 0.063 (90% CI 0.041, 0.085), SRMR = 0.020, CFI = 0.990, TLI = 0.985]. The Bollen-Stine bootstrap *p*-value of the final model was 0.106. All standardized factor loadings were adequate (Table 2; Figure 1). Internal reliability was excellent (*ω* = 0.95/*α* = 0.95).

**Table 2:** Standardized loading factors deriving from the confirmatory factor analysis of the incharge financial distress/financial well-being scale.

Item	Loading factor
1. What is your feeling about your current financial pressure?	0.81
2. What is your level of satisfaction with regard to your current financial status?	0.77
3. What is your feeling about your current financial status?	0.86
4. How many times do you worry about your ability to cover your basic monthly expenses?	0.87
5. How confident do you feel that you will be able to find the money required to cover financial emergencies of a value of 5 million Lebanese pounds?	0.76
6. How many times has the following happened to you: you wanted to go out to eat or go to a movie theater or carry out another activity and you did not because you could not afford the financial cost?	0.80
7. How many times did you find yourself trying to cope financially and live by the time you received your next financial income?	0.83
8. How much pressure do you feel with regard to your personal financial situation in general?	0.91

### Gender invariance

**Figure 2:**
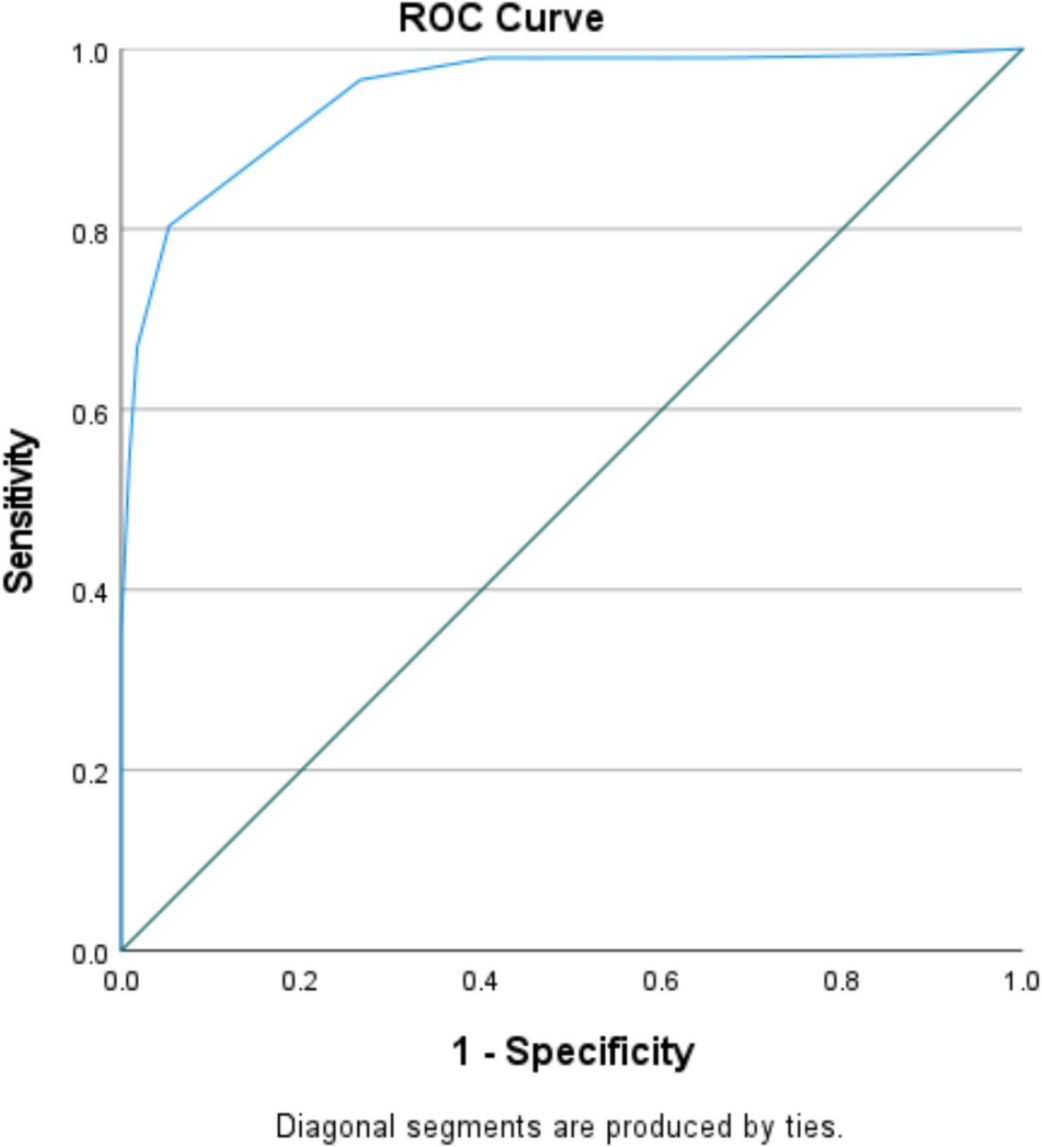
ROC curve of the single-item financial stress scale (SIFiS). Patients with high vs. low financial stress were analyzed (at a cutoff value of 40). Area under the curve of the SIFiS scale = 0.950 [0.929–0.971] (*p* < 0.001); at value = 4.50, Se = 96.6% and Sp = 73.5% and at value = 5.50, Se = 80.3% and Sp = 94.7%.

Invariance was demonstrated at the metric and scalar levels in terms of genders (Table 3). A significantly lower mean IFDFW score was found in males compared to females (47.94 ± 18.98 vs. 52.33 ± 19.39; *p* = 0.030, Cohen’s d = 0.228). When considering IFDFW item 8, no significant difference was found between males and females in terms of financial wellbeing (6.01 ± 2.67 vs. 6.53 ± 2.66; *p* = 0.062, Cohen’s d = 0.196; Figure 2).

**Table 3:** Measurement invariance of the incharge financial distress/financial well-being scale across sex.

Model	CFI	RMSEA	SRMR	Model comparison	ΔCFI	ΔRMSEA	ΔSRMR
Configural	0.985	0.055	0.031				
Metric	0.987	0.048	0.032	Configural vs. metric	0.002	0.007	0.001
Scalar	0.986	0.046	0.032	Metric vs. scalar	0.001	0.002	<0.001

CFI, Comparative fit index; RMSEA, root mean square error of approximation; SRMR, Standardized root mean square residual.

### Concurrent validity

Higher IFDFW scores were significantly associated with higher depression (*r* = 0.39; *p* < 0.001), anxiety (*r* = 0.39; *p* < 0.001), distress (*r* = 0.35; *p* < 0.001), age (*r* = 0.12; *p* = 0.017) and HCI (*r* = 0.17; *p* < 0.001). In addition, higher scores on the SIFiS were significantly associated with depression (*r* = 0.36; *p* < 0.001), anxiety (*r* = 0.39; *p* < 0.001), distress (*r* = 0.32; *p* < 0.001), age (*r* = 0.12; *p* = 0.021) and HCI (*r* = 0.12; *p* = 0.017; Table 4).

**Table 4:** Pearson correlation matrix of continuous variables.

	1	2	3	4	5	6
1. IFDFW	1					
2. SIFiS	0.90***	1				
3. Age	0.12*	0.12*	1			
4. HCI	0.17***	0.12*	−0.10	1		
5. Depression	0.39***	0.36***	−0.12*	0.06	1	
6. Anxiety	0.39***	0.39***	−0.06	0.07	0.72***	1
7. Stress	0.35***	0.32***	−0.07	0.07	0.78***	0.67***

IFDFW, InCharge Financial Distress/Well-being Scale; SIFis, Single-Item Financial Stress scale; HCI, Household Crowding Index. *p < 0.05; **p < 0.01; ***p < 0.001.

### ROC analysis of the SIFiS

The area under the curve (AUC) of the SIFiS was 0.950 [95% CI 0.929; 0.971] (*p* < 0.001). At a cutoff value of 4.50, sensitivity and specificity values were 0.966 and 0.735 respectively, whereas at a cutoff value of 5.50, they were 0.803 and 0.947, respectively.

## Discussion

The validation of the Arabic versions of the IFDFW scale and the SIFiS marks a significant advancement in assessing personal financial well-being in Arabic-speaking countries. Our analysis demonstrated strong psychometric properties for both measures, along with satisfactory concurrent validity, underscoring their robustness and reliability for use in both research and applied settings.

The Arabic translation of the IFDFW scale demonstrated that it fits the one-factor structure, as was also found in other validations (2, 17–19). This means that the scale effectively measures a unified construct of financial well-being, encompassing major subjective aspects of financial stress and satisfaction. These findings suggest that the Arabic version preserves both the theoretical and empirical integrity of the scale, supporting its application among Arabic-speaking populations. Although Robert Nielsen’s research (58) proposed a two-factor model that separates subjective (feelings about financial condition) and objective (the ability to meet expenses) financial strain, such models have generally failed to demonstrate statistical superiority or clear theoretical justification. Notably, they have been limited by issues such as low eigenvalues for the “objective” factor and the inherently subjective nature of all items within the scale. Thus, the one-factor structure remains more parsimonious and empirically robust for representing financial well-being, as further supported by our Arabic validation. A notable observation was the close relationship between Item 2 and Item 3 on one hand, and Item 6 and 7 on the other. This likely stems from semantic overlap, as each pair addresses similar themes, making them appear nearly identical to respondents and resulting in strong inter-item correlations. One could also argue that this pattern echoes the two-factor model explored by Nielsen (58), which tentatively classifies Items 1, 2, 3, and 8 under subjective financial well-being, and Items 4, 5, 6, and 7 under objective financial well-being. Furthermore, a cross-national study involving seven countries (19) highlights the multidimensional nature of financial stress in the IFDFW scale, indicating that while all items assess subjective financial stress, they may also share additional internal commonalities not fully captured by a strict one-factor model. This multidimensionality introduces measurement-related variance into single-factor models. The authors therefore recommended modified one-factor models allowing correlated errors between items—an adjustment that improved model fit in their analyses and parallels the results of our own investigation.

The Arabic version of the IFDFW had an excellent internal consistency, with both Cronbach’s alpha and McDonald’s omega coefficients at 0.95. Although a Cronbach’s alpha in the range of 0.8 to 0.9 is generally considered ideal for a short-scale measure (59), a coefficient above this range further confirms the strong psychometric properties of the scale. For context, the original IFDFW construction paper reported an identical alpha coefficient of 0.95 (2), which was also observed in a subsequent US study (19). In a multi-country validation study, the German, Italian, Dutch, Slovenian, and Spanish versions reported alpha values of 0.92, 0.91, 0.91, 0.93, and 0.93, respectively (19). In comparison, the Brazilian Portuguese version reported a Cronbach’s alpha of 0.87 (18). Such cross-cultural comparisons further underscore the high reliability of the Arabic version and suggest that future studies should explore potential cultural differences in the internal consistency of the scale.

Our findings indicate that men report relatively lower levels of financial well-being and, therefore, higher levels of distress than women. These results are inconsistent with previous research, where men generally demonstrate higher financial wellbeing than women, largely due to disparities in income and financial literacy (60–62). However, several contextual factors may explain the differences observed in our findings, particularly within the Lebanese setting.

First, men are more affected by unemployment and income losses during economic crises (63). Research suggests that men tend to equate financial well-being more directly with income, while women derive financial stability from financial behaviors and self-regulatory practices (64). Additionally, in most societies—including Lebanon—men bear the major responsibility for ensuring financial stability for their families, a societal expectation that increases their vulnerability to economic stress. This supports Gaunt and Benjamin’s findings (65), which show that traditional men experience higher job insecurity and greater levels of stress in response to financial challenges compared to traditional women, whose sense of identity is more strongly rooted in family roles and may thus be somewhat protected from economic strain.

These combined pressures, rooted in societal roles and compounded by Lebanon’s economic crisis, intensify the challenges faced by men in maintaining financial well-being.

Moreover, the SIFiS, as a single-item measure, showed no significant gender differences. This may indicate that it captures a broader, more generalized experience of financial stress, in which male and female perceptions do not significantly diverge. Alternatively, it may suggest that the SIFiS lacks the sensitivity and specificity needed to detect gender-based nuances in financial well-being influenced by cultural expectations or individual economic stressors. Further research is needed to deepen our understanding of gender differences as captured by both single-item and multi-item financial well-being measures.

Furthermore, our findings showed that higher levels of financial distress were associated with higher levels of anxiety, depression, and psychological distress, supporting the concurrent validity of our study. This was demonstrated for both the total IFDFW and the SIFiS scores. Indeed, financial strain is widely recognized as one of the major predictors of mental health problems (66). A recent systematic review (13) summarized consistent evidence of the association between financial distress and adverse mental health outcomes, including heightened anxiety, depression, and stress, which may impair decision-making and reduce quality of life. This aligns with social causation theory (12, 67–69), which suggests that stressful financial circumstances, including inadequate living conditions, malnutrition, poor health behaviors, loss of social capital, and social isolation, can contribute to the onset of new depressive symptoms or prolong existing ones. Notably, subjective financial stressors appear to weigh more than the objective measures, as the latter influence depression indirectly through an individual’s perception of financial strain (12).

## Limitations

This study has several limitations. The sample was skewed toward younger participants and females, and the use of snowball sampling may have introduced selection bias, limiting representativeness and generalizability of the findings. This method also tends to produce more homogeneous samples, as participants often recruit others from similar social circles, and it provides limited control over the recruitment process. Moreover, while the scale demonstrated strong internal consistency and structural validity, other psychometric properties were not assessed (such as test–retest reliability). Finally, this validation was conducted solely in Lebanon, underscoring the need for future studies in other Arabic-speaking populations to examine cross-cultural validity and further evaluate the scale’s psychometric properties.

### Practical implications

The Arabic IFDFW Scale has the potential to become an effective tool in research, community-based programs, and healthcare settings due to its efficiency and speed in screening for financial distress. Policymakers can utilize the scale to design targeted interventions that address financial hardship, thereby enhancing mental health and economic resilience among vulnerable populations in Arabic-speaking regions. Its adoption could support data-driven social policies, resource allocation, and population-level monitoring efforts in the Arab world, especially in contexts affected by economic instability or humanitarian crises.

Additionally, the SIFiS offers a concise and practical means of assessing financial stress, especially in settings where time and resources are limited. It demonstrated strong concurrent validity, comparable to the IFDFW. Single-item measures like the SIFiS are best suited for specific contexts, as they may be more prone to bias. They are particularly valuable when financial stress is a confounding variable or when it is important to include financial assessments without overburdening respondents. Importantly, the SIFiS is not intended to replace comprehensive tools like the IFDFW, but rather to complement them—serving as an initial screening instrument that can guide the need for more in-depth evaluation. It is especially well-suited for large-scale deployment, where time, staffing, or logistical constraints limit the feasibility of administering longer instruments. Moreover, the SIFiS may serve as a rapid screening tool in public health, humanitarian, or economic support settings, where it can be quickly deployed in large-scale assessments or triage processes to identify individuals in need of immediate support or referral.

Future public health programs across the MENA region could integrate these tools into routine data collection or rapid needs assessments, providing culturally adapted measures for identifying at-risk populations and informing targeted interventions.

## Conclusion

The Arabic version of both the IFDFW scale and the SIFiS demonstrated excellent psychometric properties, including high reliability and validity within our sample of Lebanese adults. The one-factor structure of the IFDFW supports theoretical expectations and aligns with previous validations in other languages, further confirming its value as a valid instrument for measuring financial well-being among Arabic-speaking populations. The adaptation of both measures opens the door for comparative cross-cultural studies that can deepen our understanding of how financial well-being influences societies, mental health, and quality of life worldwide.

## Data Availability

The raw data supporting the conclusions of this article will be made available by the authors, without undue reservation.
